# Post-transplantation Cyclophosphamide, Tacrolimus and Low-Dose ATG as GVHD Prophylaxis for Allogeneic Peripheral Stem Cell Transplantation for Adult Patients With Lymphoid Malignancies: A Single Arm Phase II Study

**DOI:** 10.3389/fmed.2021.630160

**Published:** 2021-03-18

**Authors:** Jie-ling Jiang, Wen-hui Gao, Li-ning Wang, Ming Wan, Ling Wang, Jiong Hu

**Affiliations:** ^1^Shanghai Institute of Hematology, Department of Hematology, Blood & Marrow Transplantation Center, Collaborative Innovation Center of Hematology, Rui Jin Hospital Affiliated to Shanghai Jiao Tong University School of Medicine, Shanghai, China; ^2^Shanghai Clinical Research Center (SCRC), Feng Lin International Centre, Shanghai, China

**Keywords:** allogeneic peripheral stem cell transplantation, graft vs. host disease, post-transplantation cyclophosphamide, lymphoid malignancies, anti-thymoglobulin

## Abstract

The PT-Cy was considered as one of the mainstay protocol for graft verus host disease (GVHD) prophylaxis. Recent study demonstrated that PT-Cy combined with other immunosuppressants could further reduce the incidence of GVHD and improve the GVHD and relapse free survival (GRFS). In this prospective phase II study, we evaluated the effect of a new GVHD prophylaxis consist of PT-Cy combined with tacrolimus and low dose anti-thymoglobulin (ATG). A total of 23 patients were enrolled including 20 patients with acute lymphoblastic leukemia (ALL) and three patients with T cell lymphoma. The median age was 29 years (range, 16~58 years). Patients with HLA-matched related donor (MSD, *n*=7) received PT-Cy combined with tacrolimus, while patients with HLA matched unrelated (MUD, *n* = 2) or haplo-identical (Haplo, *n* = 14) donor received additional ATG at 2.5 mg/kg on day 15 or day 22 after engraftment of neutrophils. As to the acute GVHD (aGVHD), only three patients developed grade I (*n* = 1) or grade II (*n* = 2) aGVHD with 100-day incidence of all aGVHD and II-IV aGVHD at 13.0 ± 5.1% and 9.1 ± 6.1% respectively. Only two patients had mild and one had moderate chronic GVHD (cGVHD), with 1-year incidence of cGVHD and moderate/severe cGVHD at 15.2 ± 8.7% and 4.6 ± 4.4% respectively. A high incidence of CMV reactivation was documented (14/16 with MUD/Haplo donor and 2/7 with MSD) with only 1 CMV disease documented. There were two EBV reactivation without post-transplantation lymphoproliferative disease (PTLD) documented. With a median follow-up of 303 days (range, 75~700 days), three patients relapsed leading to 1-year cumulative incidence of relapse (CIR) at 12.8 ± 9.2%. Only one patient died of CMV pneumonia on day 91 with both 100-day and 1-year non-relapse mortality (NRM) at 4.6 ± 4.4%. The 1-year overall survival (OS), event-free survival (EFS) and GRFS were 95.5 ± 4.4%, 82.6 ± 9.5%, and 68.0 ± 11.3% respectively. Based on Simon's stage II design, our primary data showed that the PT-Cy+tacrolimus ± ATG protocol was promising in preventing aGVHD and cGVHD, which may translate into low NRM without increased CIR. Further clinical trial with large number of patients should be warranted. This trial was registered at www.clinicaltrials.gov as #NCT 04118075.

## Introduction

Graft-versus-host disease (GVHD) is considered as a major complication after allogeneic hematopoietic stem cell transplantation (allo-HSCT) (1, 2). The post-transplantation cyclophosphamide (PT-Cy) induced immune tolerance has been translated from laboratory studies into clinical practice and clinical data confirmed that PT-Cy can be considered as the mainstay strategy for GVHD prophylaxis in allo-HSCT of HLA-matched related (MSD), unrelated (MUD) and haplo-identical (Haplo) settings (3–8).

Though there are plenty clinical reports of PT-Cy for patients with acute myeloid leukemia (AML) and myelodysplasia syndrome (MDS), the studies focusing on lymphoid malignancies are still limited (9). In our previous phase II clinical trial, we reported that the incidence of grade II-IV acute GVHD (aGVHD) and moderate to severe chronic GVHD (cGVHD) were as high as 39% and 24% respectively in adult patients with lymphoid malignancies receiving PT-Cy in combination with cyclosporine as GVHD prophylaxis and using mobilized peripheral blood stem cells (PBSCs) as graft (10). Our data were consistent with the EBMT registry study which demonstrated that in MSD/MUD settings, acute lymphoblastic leukemia (ALL) and unrelated donor transplantation were associated with higher incidence of grade II-IV aGVHD, while graft of PBSCs was an important risk factor for overall cGVHD and extensive cGVHD (ext cGVHD) (11). Of note, the analysis also indicated that PT-Cy combined with two immunosuppressants or with *in vivo* T cell depletion agents (TCD) significantly reduced the risk of ext cGVHD, which remained as a key prognostic factor for an improved GVHD and relapse-free survival (GRFS).

To further reduce the incidence of GVHD and improve GRFS, we evaluated a new GVHD protocol of PT-Cy followed additional tacrolimus and low-dose anti-thymoglobulin (ATG) in patients with lymphoid malignancies undergoing first allo-HSCT with mobilized peripheral blood as graft.

## Patients and Methods

### Patients and Eligibility Criteria for Allo-HSCT

This was an investigator-initiated, prospective, non-randomized, single-arm phase II clinical trial (NCT 04118075). The study was approved by the Human Ethics Committee of the Rui Jin Hospital and was in accordance with the Declaration of Helsinki. The study was conducted in the Blood and Marrow Transplantation Center of Rui Jin Hospital, Shanghai Jiao Tong University School of Medicine.

The inclusion criteria were as follows: (1) adult patients (18 to 60 years old) with acute lymphoid malignancies including ALL or various non-Hodgkin's lymphoma; (2) disease status at transplantation including first or second complete remission (CR1 or CR2) for ALL; CR, partial remission (PR) for high-grade lymphoma or any stage of low-grade lymphoma; (3) patients received first allo-HSCT from MSD, MUD or Haplo donor; (4) patients with performance status ≤2 (ECOG score) with acceptable renal and hepatic function (serum creatinine ≤133 μmol/L, serum bilirubin ≤34 μmol/L, serum glutamic-pyruvic transaminase <3 times of the upper normal limit), cardiac left ventricular ejection fraction ≥50% and normal pulmonary function tests; (5) all patients provided written informed consent before allo-HSCT.

### Conditioning and GVHD Prophylaxis Regimen

All patients received fludarabine (FLU) 30mg/m^2^/ d from day−7 to day−3, etoposide 400 mg/m^2^/d from day−7 to day−6 and intravenous busulfan (BU) at 1.6 mg/kg every 12 h for 6 doses over 3 days (from day−5 to day−3). BU was administered by infusion over 3 h and the dose of BU was based on actual body weight (ABW) or adjusted ideal body weight [AIBW, AIBW=ideal body weight (IBW)+25%(ABW-IBW)] for overweight patients. Granulocyte colony-stimulating factor (G-CSF) mobilized PBSCs were infused on day 0. GVHD prophylaxis consisted of cyclophosphamide 50 mg/kg on day 3 and day 4 followed by tacrolimus 0.5 mg/kg twice daily starting on day 5 adjusted with target therapeutic trough level of 5~15 ng/ml. Single dose of 2.5 mg/kg ATG was given on day 15 after documentation of neutrophil engraftment in patients undergo MUD or Haplo transplantation. In case of delayed ANC engraft, the dose of ATG was postponed for 7 days to day 22 or afterwards likewise. To avoid the impact of donor lymphocyte infusion (DLI) on the development of aGVHD, no prophylactic DLI was allowed within day 100. DLI was only allowed in case of positive measurable residual disease (MRD) or bone marrow relapse after day 100. The conditioning and GVHD prophylaxis regimens were presented in Table 1.

**Table 1:** Conditioning regimen and GVHD prophylaxis.

**Drug**	**Dosages**	**Days**
Fludarabine	30mg/m^2^ daily	D-7 to D-3
Etopside	400mg/m^2^daily	D-7 to D-6
Busulfan	1.6mg/kg q12h	D-5 to D-3
PBSC	/	D 0
PT-Cy	50mg/kg daily	D3 to D4
tacrolimus	0.5mg/kg q12h p.o.	D5 forward
ATG	2.5mg/kg once	D15 or D22^*^

* *In case of transplantation from MUD or Haplo donor, ATG (2.5 mg/kg) was given on D15 if ANC engraftment was documented. In patients with late ANC engraftment, ATG was delayed to D22 or weekly basis afterward*.

### Supportive Care

Acute and chronic GVHD were diagnosed and graded according to standard guidelines (12, 13). In case of grade I–IV aGVHD, the first line treatment was methylprednisolone 1~2 mg/kg/day (i.v.) alone or combined with basiliximab. If no response, ruxolitinib was added as second line treatment. For cytomegalovirus (CMV), prophylaxis ganciclovir was given on day−9 to day−2. After transplantation, pre-emptive treatment ganciclovir was based on weekly monitoring and given in case of CMV DNAemia level >1 × 10^4^ copy/ml. For Epstein-Barr virus (EBV) associated post-transplantation lymphoproliferative disease (PTLD), pre-emptive rituximab was also given based on weekly monitoring of EBV DNAemia when EBV DNA level was over >1 × 10^5^ IU/ml. For prophylaxis of pneumocystis, sulfamethoxazole was given every other day from engraftment to 100 days after transplantation. As to the prophylaxis of invasive fungal disease (IFD), risk adapted escalation strategy was used. Specifically, all patients without previous probable or proven IFD received fluconazole (i.v.) as IFD prophylaxis in the laminar airflow unit. After patients were discharged from the BMT units, fluconazole (p.o) was given to day 100 in patients undergoing transplantation from MSD without aGVHD. While for all patients with unrelated or haplo-donor and all patients developed aGVHD requiring steroid treatment, the prophylaxis was escalated to voriconazole or posaconazole until day 100. For patients with previous documented probable or proven IFD, caspofungin or voriconazole was given in the BMT unit as secondary prevention and voriconazole or posaconazole was used sequentially until day 100. All other supportive care was according to the standard protocol of Rui Jin Hospital.

### Study Endpoints

The primary endpoint of the study was the incidence of grade II to IV aGVHD at day 100. Secondary endpoints included the median time to the recovery of neutrophil or platelet, 100-day overall aGVHD and non-relapse mortality (NRM), 1-year overall cGVHD and moderate to severe cGVHD and 1-year overall survival (OS), event-free survival (EFS), cumulative incidence of relapse (CIR), NRM and GRFS. For OS, the failure was defined as death from any cause. For EFS, failure was defined as documented event of either disease relapse or death from any cause. Patients were considered to have died of NRM if there was no evidence of disease relapse or progression before death. Relapse was defined as >5% blasts in bone marrow (BM) examination or extramedullary relapse documented by biopsy in patients with ALL and positive PET-CT scan followed by biopsy in patients with lymphoma. GRFS was defined as patients without the following events including relapse, graft failure, occurrence of III-IV aGVHD and moderate or severe cGVHD.

### Sample Size Estimation and Statistical Analysis

The study was based on assumption that the addition of tacrolimus and low dose ATG to PT-Cy decreased the incidence of grade II-IV aGVHD at day 100 to an optimal level of 15% and 40% as unacceptable high as in our previous prospective study with PT-Cy and cyclosporine. The goal of enrollment was set at 23 patients based on Simon's stage II design permitting early stop of trial if 15% was unlikely to achieve (14). With an optimal design, nine patients were enrolled in stage I. If six patients had grade II–IV aGVHD at day 100, the trial should be stopped early for futility. Otherwise, 14 more patients should be enrolled in stage II (as shown in Supplementary Table 1).

The cumulated incidence of aGVHD was calculated using relapse, death of any causes as competing risks (15). The incidence of cGVHD, relapse and NRM was summarized using cumulative incidence estimate accordingly. The OS, EFS and GRFS were calculated using the Kaplan-Meier method (16). Data for patients who were alive in CR were censored at last follow-up on September 30, 2020. The statistics was performed by SPSS and R software at Shanghai Clinical Research Center.

## Results

### Patients and Characteristics

A total of 23 patients were enrolled in the study. All patients have been followed-up for a median of 303 days (range, 75~700 days). Demographic data for these patients were summarized in Table 2. The median age was 29 years (range, 16~58 years). Twenty patients were ALL including 7 with T-lineage (T-ALL), 12 with B-lineage (B-ALL) and 1 with mixed lineage ALL (Mix-ALL). Among B-ALL, 6 patients were Philadelphia chromosome positive ALL (Ph^+^ ALL) and received tyrosine kinase such as imatinib or dasatinib before and after allo-HSCT. All patients with ALL were in CR1. Three patients with lymphoma were also enrolled, including one with T-lymphoblastic lymphoma in PR, one with refractory Sezary syndrome and 1 with ALK^+^ anaplastic large cell lymphoma (ALCL) in CR3. The donors included seven from MSD, two from MUD and 14 from Haplo. In this series, the pre-transplantation status of serum CMV was positive in all patients and their donors.

**Table 2:** Patient characteristics.

**Characteristics**	**Values/n**
Age, median (range) years	29 (16~58)
**Sex**	
Male	19 (82.6%)
Female	4 (17.4%)
**Diagnosis**	
ALL	20 (87.0%)
T-lineage	8
B-lineage	12 (ph^+^ 6; ph^−^ 6)
Lymphoma	3 (13.0%)
Sezary syndrome	1
ALCL	1
T-LBL	1
**Disease status**	
CR1	20 (87.0%)
PR/CR2 or beyond	3 (13.0%)
**Donor type**	
MSD	7 (30.4%)
MUD	2 (8.7%)
Haplo	14 (60.9%)
**GVHD prophylaxis**	
PT-Cy+FK506	7 (30.4%)
PT-Cy+FK506+ATG	16 (69.6%)
**CMV serum status**	
Donor^+^/Recipient^+^	23 (100%)

### Engraftment and Chimerism

For all 23 patients, the median number of mononuclear cells and CD34^+^ cells infused was 6.7×10^8^/kg (range, 2.3~15.7×10^8^/kg) and 4.7×10^6^/kg (range, 2.3~11.6×10^6^/kg), respectively. Neutrophil engraftment occurred in all 23 patients at a median of 14 days (range, 10~20 days), and the median time of platelet recovery was 24 days (range, 9~67 days) in 22 patients. The chimerism analysis of total mononulear cells and CD3^+^T cells in bone marrow was performed in all patients on day 28 based on PCR of short tandem repeat (STR) unique for donor or recipient. Among them, 22 patients achieved full donor chimerism (≥99%) while one patient with haplo donor failed to achieve full donor chimerism (<60%) and quickly developed pancytopenia with loss of donor chimerism and regarded as primary graft failure. The very patient underwent salvage allo-HSCT from MUD donor and remained alive in CR at last follow-up.

### Acute GVHD and Chronic GVHD

For GVHD prophylaxis, ATG was given to all patients transplanted from MUD or Haplo donor on day 15 (*n* = 9) or day 22 (*n* = 7) according to the day of ANC engraftment. As to the aGVHD, only one patient developed grade I aGVHD (skin, stage II) and two patients developed grade II aGVHD (skin, stage I~II; gut, stage I, defined as diarrhea <1,000 ml/day). There was no grade III-IV aGVHD documented. At day 100, the cumulative incidence of all grade aGVHD and II–IV aGVHD were 13.0 ± 5.1% and 9.1 ± 6.1% respectively. As to the cGVHD, only two patients had mild cGVHD involving skin or liver respectively, while another patient developed moderate cGVHD, which was characterized by involvement of skin, liver and mucosa. During the follow-up, no patient had documented abnormal pulmonary function, therefore no bronchiolitis obliterans (BO) was diagnosed. The 1-year cumulated incidence of cGVHD and moderate/severe cGVHD were 15.2 ± 8.7% and 4.6 ± 4.4% respectively.

### Infection and Other Complications

During the follow-up, a total of 16 patients experienced CMV reactivation. The median time to documented CMV DNAemia was 42 days (range, 30~109 days) with median CMV level of 15 (2~170)×10^3^ copies/ml. Six patients with low viral load (<1×10^4^ copies/ml) recovered spontaneously while ganciclovir was given in 10 patients. Among them, two patients received Haplo transplantation developed pancytopenia after ganciclovir treatment and eventually had secondary graft failure without evidence of relapse on day 60 and day 135 respectively. These two patients received subsequent 2nd allo-HSCT from MUD donor and both remained alive in CR at the last follow-up. CMV disease (pneumonia) was documented in one patient on day 55. The very patient suffered from respiratory failure and received combination therapy of ganciclovir, foscarnet, broad-spectrum antibiotics and assisted ventilation before eventually died on day 91.

As to the EBV infection, there were 2 patients with EBV DNAemia at low level of 1 and 5×10^3^ IU/ml, which remained stable without any PTLD associated symptoms and eventually became negative in the subsequent follow-up.

There were two patients with previous documented IFD including one with proven candidiasis of liver and spleen and one with probable invasive pulmonary aspergillosis (IPA) before transplantation. With secondary prophylaxis, the patient with candidiasis had an episode of candida blood stream infection, which was controlled quickly with addition of amphotericin B to caspofungin. And the patient with IPA remained stable during the process of HSCT. A total of five patients required empirical anti-fungi treatment because of persistent febrile neutropenia with sufficient antibiotic treatment. With IFD work-up, only one patient was diagnosed as probable IPA.

As to other complications, mild hemorrhagic cystitis (HC) was documented in 4 patients and all responded well to hyperhydration with diuretics. All patients who developed HC were in the ATG group. Of note, 2 of them developed HC around day 6 (just after PT-Cy) and the other two occurred around day 35 (after ATG treatment). There was no sinusoidal obstruction syndrome (SOS) and grade III–IV liver toxicity ever documented in all 23 patients. One patient developed Guillian-Barre syndrome on day 45 with unknown cause responded well to high-dose intravenous immunoglobulin (IVIG) and fully recovered afterwards.

### Overall Outcome

**Figure 1:**
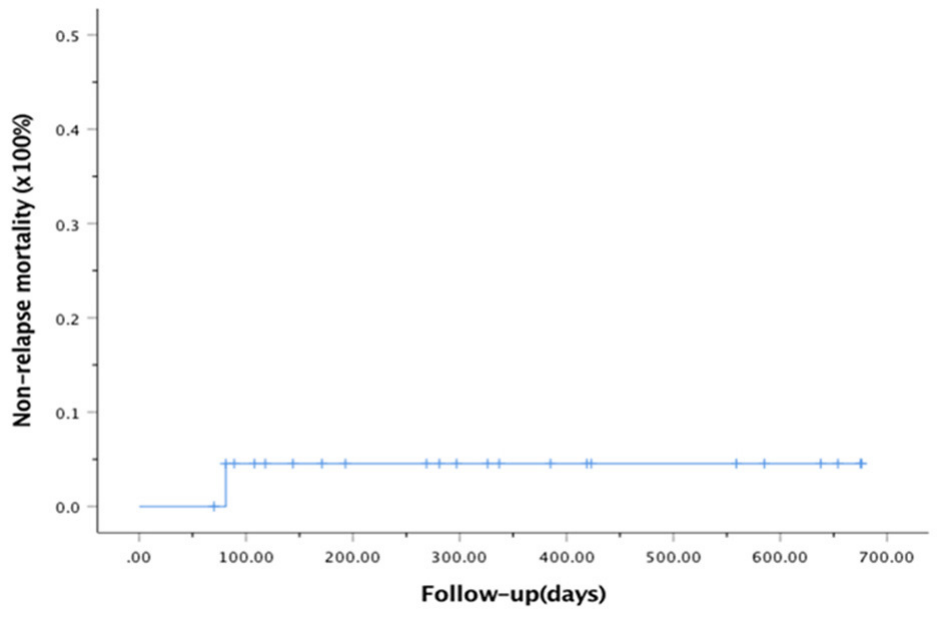
Analysis of non-relapse mortality (NRM).

**Figure 2:**
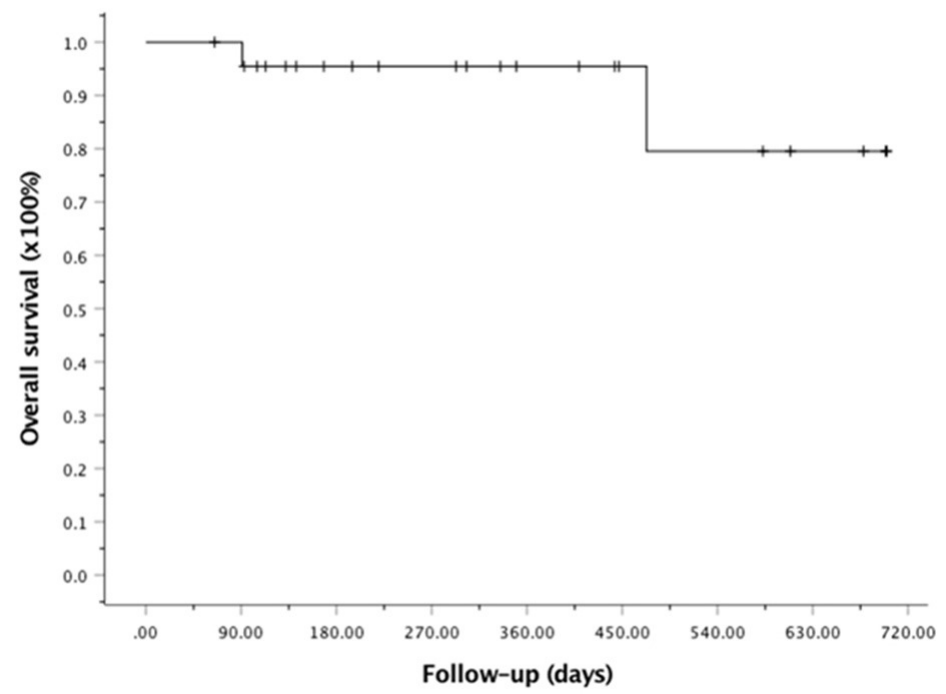
Kaplan-Meier analysis of overall survival (OS).

**Figure 3:**
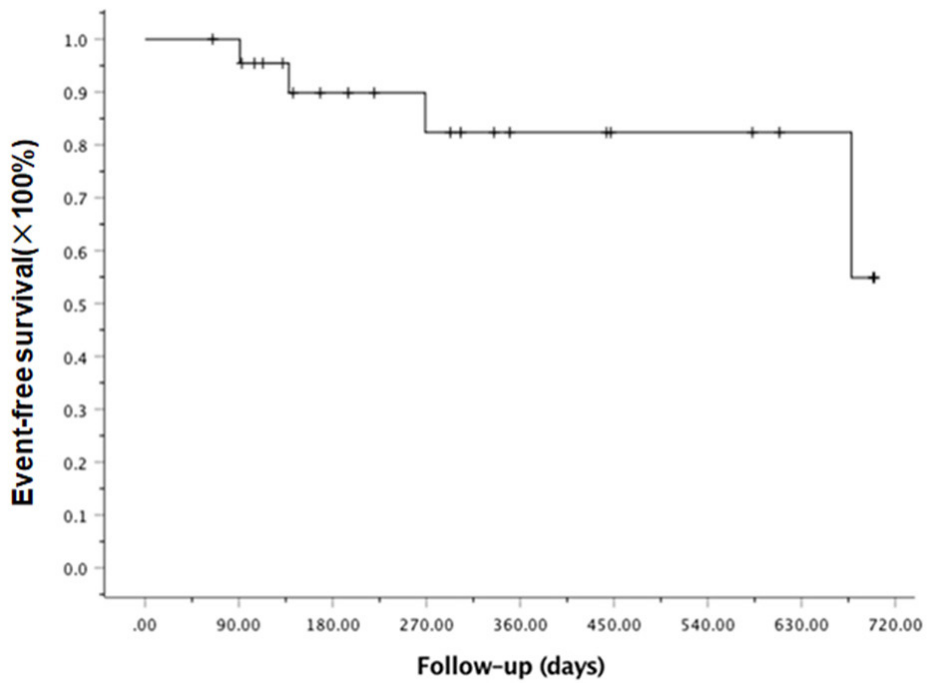
Kaplan-Meier analysis of event- free survival (EFS).

**Figure 4:**
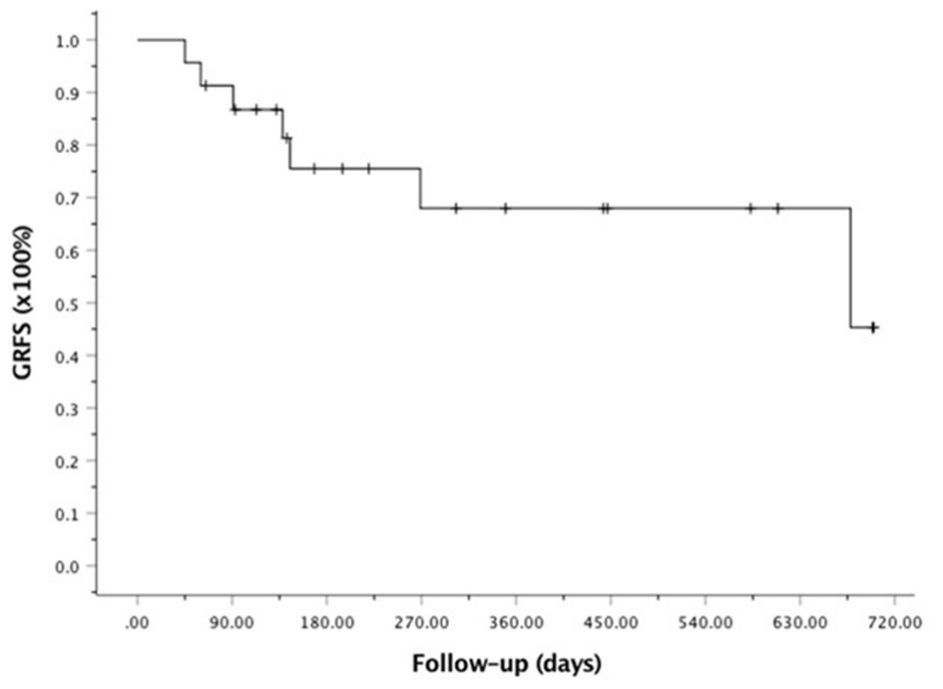
Kaplan-Meier analysis of GVHD and relapse-free survival (GRFS).

At the last follow-up, one patient died of CMV pneumonia on day 91, making the 100-day and 1-year NRM both at 4.6 ± 4.4% (as shown in Figure 1). A total of 3 patients with T -ALL experienced relapse on day 138 (BM), 269 (extramedullary) and 678 (BM) respectively, making the 1-year CIR at 12.8 ± 9.2%. The very patient who relapsed on day 138 received DLI on day 151 and 182 following low-dose chemotherapy (CAG regimen). He achieved CR2 together with grade III aGVHD after DLI. Unfortunately, the patient relapsed again 3 months later and eventually died of disease. The other two patients were still under treatment of relapse and 19 patients remained alive in CR. The 1-year OS and EFS were 95.5 ± 4.4% and 82.6 ± 9.5% (as shown in Figures 2, 3), respectively. Overall, a total of 16 patients remained alive and free of relapse, graft failure, III-IV aGVHD and moderate to severe cGVHD with 1-year GRFS of 68.0 ± 11.3% (as shown in Figure 4).

## Discussion

The PT-Cy alone or in combination with other immunosuppressants was considered as one of the mainstay of GVHD prophylaxis strategies in various hematological malignancies (1, 2, 17–19). In a retrospective analysis from Acute Leukemia Working Party of the EBMT (AWLP-EBMT) with 423 patients transplanted in MSD and MUD setting, the diagnosis of ALL (vs. AML, *p* = 0.008) and PBSCs as graft (vs. BM, *p* = 0.01) were associated with higher incidence of II-IV aGVHD or ext cGVHD respectively. As to the GVHD protocol (PT-Cy alone or combined with other immunosuppressants), it did not impact the incidence of II–IV aGVHD, while the incidence of ext cGVHD was significantly higher for PT-Cy alone or with one immunosuppressant (18 or 20%) compare to only 8.5% for PT-Cy in combination with 2 immunosuppressants (*p* = 0.02). More interestingly, adding ATG to PT-Cy was independently associated with reduced risk of ext cGVHD (*p* < 0.001). As to the outcome of allo-HSCT, the PT-Cy with 2 additional immunosuppressants did not impact the incidence of relapse (*p* = 0.57) and NRM (*p* = 0.05), but improved both OS (*p* = 0.03) and GRFS (*p* = 0.007). Besides, adding ATG to PT-Cy did not impact the relapse rate (*p* = 0.61) in multivariate analysis (11).

More recently, clinical study demonstrated that ATG (2.5 mg/kg on day-1 or day-2 to−1) added as part of pre-transplantation conditioning combined with PT-Cy decreased aGVHD (12 vs. 22%, *p* = 0.029), reduced NRM (8 vs. 23%, *p* = 0.005) and lead to an improved OS (79 vs. 69%, *p* = 0.029) at 1 year compared to the no ATG group (20). In another retrospective study with thiotepa based conditioning and PBSCs as graft in Haplo setting, pre-transplantation ATG (1 mg/kg from day−6 to day−2) and the PT-Cy were used as GVHD prophylaxis. It showed that the 2-year cumulative incidences of III-IV aGVHD, severe cGVHD, NRM and relapse were 16, 16, 26, and 26%, respectively, which compared favorably with those of other reports using BM as graft (21).

Based on the above data, we designed a new GVHD prophylaxis regimen with PT-Cy combined with tacrolimus and low-dose ATG (2.5 mg/kg) after ANC engraftment (on day 15 or day 22). Interestingly, our data demonstrated that low incidence of overall II-IV aGVHD (9.1 ± 6.1% at 100-day) could be achieved and even no III–IV aGVHD observed. Though the follow-up was still limited, we observed a trend of low incidence of cGVHD and moderate/severe cGVHD (4.6±4.4% at 1-year). Moreover, the lower incidence of GVHD was translated into lower 100-day NRM (4.6 ± 4.4%). The overall outcome concerning aGVHD/cGVHD and NRM was significantly better than our previous study using PT-Cy with cyclosporine only in the setting of lymphoid malignancies undergoing allo-HSCT with similar myeloablative conditioning and PBSCs as graft (10).

ESQUIROL et al. declared that single-agent of tacrolimus after PT-Cy was a valid option for haploidentical transplantation in adults with hematological malignancies (22). However, the cumulative incidence of grade II–IV and III–IV aGVHD were 23 and 14%, respectively, and the incidence of ext cGVHD was as high as 22% in their study. In another study, Carnevale-Schianca et al. reported low aGVHD incidence of 17% and cGVHD incidence of 7% using PT-Cy, tacrolimus and mycophenolate mofetil (MMF) as GVHD prophylaxis, though 46% of enrolled patients were diagnosed as AML and no patient was transplanted from haploidentical donor (23). These studies and our recent study were consistent with the AWLP-EBMT study, which demonstrated that the incidence of GVHD was significantly higher for PT-Cy alone. Thus, tacrolimus, MMF, ATG or other immunosuppressants should be considered as combination with PT-Cy.

Mobilized PBSC as graft tend to have higher risk of cGVHD compare to bone marrow even with PT-Cy strategy. In our previous study using PT-Cy and cyclosporine, though cGVHD was reduced compare to our historical control using cyclosporine, MMF and MTX regimen, moderate/severe cGVHD including BO was documented. With low dose ATG added to PT-Cy/tacrolimus, no cases of BO documented yet. Longer follow-up should be anticipated to confirm this observation. Thus, we speculated that the addition of low dose ATG may benefit patients undergoing PBSCT and may had limited benefit or no benefit in BMT setting, since BM was associated less cGVHD, Of course, it need to be tested.

Chronic GVHD was considered to be associated with the potential of GVL effect so that may be of benefit to relapse control. The analysis of EBMT registry data demonstrated that addition of ATG may increase the potential relapse rate after allo-HSCT for both ph^+^ or ph^−^ ALL, but may improve GRFS (24, 25). Other report suggested that low-dose ATG added to PT-Cy protocol may have limited effect on relapse in Haplo setting (20). In our study, though with limited follow-up time, the early incidence of relapse (1-year) was not aggravated with addition of ATG for lymphoid malignancies, but longer follow-up was required to confirm. Nowadays, more emphasis has been put on post-transplantation treatments to prevent diseases relapse. For example, TKIs, hypomethylation agents and/or immune modulation therapy such as DLI or interferon, are all candidates for using, especially for AML patients. Thus, low incidence or no cGVHD may not be necessarily associated with increased relapse. A new clinical trial using the same GVHD prophylaxis in AML patients is undergoing in our center, which is focus on the relapse rate with low-dose decitabine as maintenance therapy after transplantation.

As to the safety of the protocol, we observed a quite high incidence of CMV reactivation particularly in patients transplanted from MUD or Haplo donor (14/16) vs. MSD (2/7, *p*<0.001). This high incidence of CMV reactivation may attribute to late addition of ATG even with low dose of 2.5 mg/kg due to its direct immune suppression and/or delay of CMV specific immune reconstitution (26). In further clinical study, reducing the dose of ATG or more effective CMV prophylaxis such as use of letermovir after ATG should be considered (27). Of note, we also observed two cases of secondary graft failure. Since no patient had graft failure immediately after ATG treatment and pancytopenia occurred after weeks of ganciclovir treatment, we speculated that low-dose ATG might increase the risk of CMV infection and indirectly increase the risk of secondary graft failure. However, further clinical trial with large number of patients should be warranted especially with letermovir prophylaxis.

As we know, this is the first clinical trial reported to evaluate the role of low-dose ATG added after PT-Cy in the setting of allo-HSCT with PBSCs as graft in lymphoid malignancies. The limitation of the study was the number of patients enrolled. Since the aim of Simon's stage II design was to reject the protocol early when it was unlikely to achieve optimal response, our initial data supported that PT-Cy + tacrolimus ±ATG was potentially effective in reduction of aGVHD/cGVHD and NRM. Further clinical trial with large number of patients should be warranted.

## Data Availability Statement

The original contributions presented in the study are included in the article/Supplementary Material, further inquiries can be directed to the corresponding author/s.

## Ethics Statement

The studies involving human participants were reviewed and approved by the Human Ethics Committee of the Rui Jin Hospital. The patients/participants provided their written informed consent to participate in this study.

## Author Contributions

JH and LW designed the clinical trial, revised the manuscript. J-lJ and W-hG collected the clinical data, and wrote the manuscript. L-nW also helped to collect the clinical data. MW did the statistical analysis. All authors contributed to the article and approved the submitted version.

## Conflict of Interest

The authors declare that the research was conducted in the absence of any commercial or financial relationships that could be construed as a potential conflict of interest.
