# Supplementary material for: Post-transplantation Cyclophosphamide, Tacrolimus and Low-Dose ATG as GVHD Prophylaxis for Allogeneic Peripheral Stem Cell Transplantation for Adult Patients With Lymphoid Malignancies: A Single Arm Phase II Study
Source: Front Med (Lausanne). 2021 Mar 18;8:630160. doi: 10.3389/fmed.2021.630160 (PMC8012531; doi:10.3389/fmed.2021.630160)
Supplement: Supplementary file 1 [file Table_1.docx]

Supplemental Table-S1

| N | n1 | r1 | r2 | Type 1 Error | Power | EN0^ | Probability of early stopping | Interval for w# | Model |
| --- | --- | --- | --- | --- | --- | --- | --- | --- | --- |
| 20 | 7 | 4 | 15 | 0.0470 | 0.8040 | 12.5 | 0.5801 | [0.0666,1] | Minimax |
| 23* | 9 | 6 | 17 | 0.0418 | 0.8012 | 12.2 | 0.7682 | [0,0.0665] | Optimal |

* In this study to evaluate the efficacy of PT-Cy + tacrolimus ±ATG regimen in GVHD prophylaxis, the good response is defined as no grade II-IV aGVHD documented within Day 100 after allo-HSCT. The target grade II-IV aGVHD is 15% compare to historical control of 40%.

According to the Simon’s Stage II design, N is the total number of patients should be enrolled; n1 is the number of patients accrued during stage I. With an optimal design, a total of 9 patients should be enrolled in stage I. If 6 (r1) or fewer patients had good response (no grade II-IV aGVHD at Day100), the trial is stopped early for futility. Otherwise, study entered to Stage II and another 14 (N-n1) patients should be enrolled. At the end of study, the null hypothesis will be rejected if 18 (r2+1) or more patients achieved response (no grade II-IV aGVHD at Day 100) and further confirmation study was warranted. If only 17 or fewer had good response, then no further investigation of this protocol is warranted. This design yields a type I error rate of 0.0418 and power of 0.8012 when the true grade II-IV aGVHD rate at Day 100 is 15%.

^ EN0 is the expected sample size for the trial when response rate is p0;

# Interval for w is the set of values w such that the design minimizes w * n + (1 – w) * EN0
